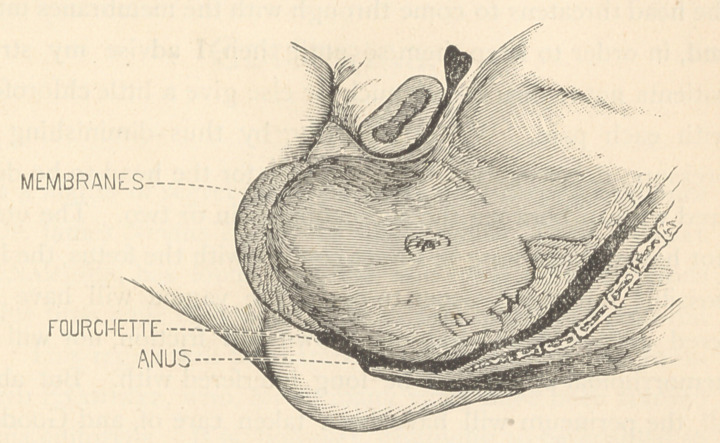# Functions of the Membranes in Labor

**Published:** 1885-03

**Authors:** Henry T. Byford

**Affiliations:** 3100 Forest Ave.; Chicago


### ARTICLE III.

### FUNCTIONS OF THE MEMBRANES IN LABOR.

HENRY T. BYFORD, M. D., OF CHICAGO.

[Read before the Chicago Gynecological Society, Feb. 20th, 1885.]

"It is hardly an exaggeration to state that the greater portion of the sins of midwifery practice are committed in the management of natural labor," says Lusk, in his "Science and Art of Midwifery," page 202, and on page 238, again, "The *os externum* is usually torn, especially upon the sides, and the thickened labia roll outwards."

Other reliable authorities teach that one-fifth of the number of mothers suffer laceration of the perineum at the birth of the first child. Hæmorrhoids are said, by Barnes, and are generally considered, to be common results of ordinary labor. With such facts before us, we may well wonder, with the late Professor Gross, "why the Almighty did not create, simultaneously with woman, a competent gynecologist to meet these inevitable evils." It seems, indeed, like a reproach upon Him, the crowning work of whose intelligence was the creation of woman, that she should be the most poorly prepared of all beings for the reproduction of her kind. Was it always thus, or was child-bearing originally a "physiological phenomenon, not beyond the power of a healthy woman to patiently endure?"

If civilization be at fault, then we must again admit, with Professor Gross that "the sooner we get rid of this civilization the better will it be for our poor wives and daughters as procreative beings." Or, if the above quoted "sins of midwifery practice" have anything to do with these evils, it is time we were abandoning some of the things we have learned in the past six thousand years, and that the management of natural labor, especially the care of the perineum, were so taught as to come within the mental grasp of the ordinarily intelligent medical graduate.

The departure from the methods now taught, upon which I wish to get an expression of your opinion, is the preservation of the membranes from rupture until they dilate, or aid in dilating, the vulva, as they are sometimes observed to do when they are unusually tough and the child is born with a "caul." I shall first say a few words about the possibility and probability of such a procedure and afterwards about the utility.

According to the present obstetric teaching, and practice, I believe, the patient in labor is encouraged to go about the room until near the end of the first stage, thus, whether purposely or not, stimulating the uterus to contraction and adding gravitation to the other forces that bear upon the membranes in the cervix. This practice, with subsequent pulling at the os with the finger, or pressure upon the abdomen whenever the pains lag a little, or both, often has for its effect the discharge of the amniotic fluid, at, or near the end of, the first stage. If, however, pursuing an opposite course, we put the patient to bed upon her side as soon as, or soon after, the os commences to dilate; and if the pains are more severe than can be easily borne (as they are apt to be in highly civilized communities), we give an opiate, or other anodyne, to keep her comfortable, the presenting bag will be relieved of the weight of the abdominal contents, and saved from the more intense and frequent action of expulsive powers that accompanies an erect, or semi-erect, position.

After the os is widely dilated, and the membranes receive but little support from the cervix, they will be less liable to rupture if we put the patient upon her back during uterine action. The head will be better able to form a ball valve, and thus take part of the pressure from the presenting pouch, viz.; as much as is required to overcome the elasticity of the ring of the cervix against which the descending head impinges. Now, after the os is widely dilated, when the resistance to the descent of the head is small, and the consequent strain upon the protruding membranes great, a change occurs which invalidates the reasoning by which some of the German authors, would make their rupture, or the bringing of labor to a stand-still inevitable at or near the complete opening up of the uterine neck. The membranes, being loosened from the lower segment of the contracting uterus, are pushed down through the dilated cervix along with the head, instead of being merely stretched; they come in contact with the undilated vagina and are supported by it; and, as the whole parturient canal is converted into one cavity, the head follows the bag of waters down to the vulva, just as it followed it down to the external os, aiding in the dilatation, and sharing the pressure from behind. The second stage of labor becomes a kind of repetition of the first, the dilatation of the vagina and vulva being an analogue of that of the cervix and external os.

The Germans divide the expulsive powers of the uterus into two factors, the "internal uterine pressure" and the "form-restitution power." The "internal uterine pressure" is the uniform pressure to which the whole ovum is subjected; the "form-restitution power" is the shortening of the uterus from above downwards and from side to side. When the os is fully dilated, the "internal uterine pressure" forces the lower end of the bag of membranes down into the vagina because that is the direction of the least resistance, but the "form-restitution power," by shortening the uterus from above downwards, forces the head down against the cervix, aided by the shortening of the transverse diameter, which tends to keep the body of the child from being displaced sideways. Now, all of these forces are acting in a downward direction, and the os being dilated, and the membranes loosened, what is to prevent them from descending to the vulva?

The membranes, with such management, are subjected to no more strain during the second stage of labor than they are under the prevalent mode of treatment, during the first stage. In fact the pressure upon the membranes under the ordinary start-on-the-run management, is not usually sufficient to rupture them as early as is commonly supposed. In my experience they have either ruptured at or near the beginning of labor or else persisted after the os has been dilated. Students are taught to rupture at the end of first stage; and nearly all of the physicians I have consulted usually find it necessary to do the same. Indeed, recent graduates are apt to betray a thrill of pride at the recital of the number of times they have successfully performed this hair-pin obstetric operation.

Playfair states, page 251, that a child would more frequently be born with a caul " * * were it not the custom of the accoucheur to rupture the membranes artificially as soon as the os is completely opened up * * " Leishman, in the second American edition of his "System of Midwifery," 1875, page 261, says, in describing labor: "As the termination of the first stage approaches, the protrusion of the bag of the membranes becomes more and more marked; and as at the same time the pains usually become more violent, it often excites our astonishment that rupture is so long delayed." The only really astonishing thing is that one of such profound learning should be so often astonished at the same occurrence.

Scanzoni states, page 338, vol. I.: "For it often happens that the child is expelled immediately or shortly after the rupture of the membranes," an occurrence that must either be the result of a dilatation of the vagina by the membranes, or else an already dilated condition. In those cases where the soft parts are flabby and can offer no resistance to the passage of the head, the preservation of the membranes is, of course, unimportant; and as the vagina affords no support, they are liable to rupture at any moment after the os is opened up. But in primipara and multipara, whose soft-parts had returned to a natural condition after previous confinements, every attention, even the slightest, that makes labor safer and easier, is of great importance.

Naegele not having been obliged, in his day, to maintain his reputation for learning by an analysis and theoretical discussion of the profusely titled powers and pressures of the gravid uterus, made himself quite comprehensible by simply advising against rupture of the membranes until their appearance externally at the vulva.

The following is a fair sample of a large proportion of obstetric cases in my practice since I have adopted this plan:

Mrs. M——, a healthy, robust, Canadian primipara, twenty-five years old, sent for me at 5 A. M. I found her suffering intensely with very frequent and very painful uterine contractions, which had commenced about six hours before. The os uteri was dilated to about the size of a silver twenty-cent piece, and in another hour to, perhaps, the size of a silver quarter, of a dollar, and the pains continued as before. I prescribed fifteen minim doses of McMunn's Elixir of Opium, to be repeated every hour until easier, and left her for two hours. I returned to her soon after she had taken the third and last dose and found her dozing between pains and not complaining at all. I

ruptured the membranes after they had protruded through the vulva and delivered her at 10 A. M. No unusual symptoms occurred then or afterwards, except that she (I use her own expression) "had the baby almost without any pain."

When the membranes are ruptured at the end of the first stage, there results, after a temporary rest, a rapid and very great change in the character of the labor, but when they are preserved from rupture it is impossible to tell, without the finger in the vagina when the first stage ends and the second begins. The torment that belongs to the stretching of the cervix subsides, and the labor gradually assumes a straining character, but only at the last moment, after the vulva is dilated to half or two-thirds of the size necessary for the exit of the head, is that severe suffering felt, of which primipara are otherwise wont to complain as soon as the head commences to bulge the perineum, or even after. The vagina is filled with a tumor so smooth and soft that its presence cannot be felt by the patient at any one point, so elastic that a light finger touch indents it, and yet the whole power of almost every uterine muscular fiber, bears efficiently upon it.

'Tis strange that obstetric science can teach the deliberate breaking up of this simple process of nature and the substituting of an unnatural and artificial one, by which nearly all of the fibers of the lower half of the uterus operate only to contuse its own structure against the bare parts of the fetus without materially aiding in its descent, while the fibers of the fundus jam the hard, rough, inelastic, and comparatively unyielding head, down through the vagina against the perineal center, bulging and stretching the perineum, according to Lusk, "from four to five inches in its antro-posterior direction," and all of this without any provision for a gradual dilation of the vulva, or of making its axis, before delivery of the head, correspond with the axis of the pelvis.

After Schroeder.

Let us glance, for a moment, at the mechanism of dilatation of the perineum and vulva by means of the membranes: When the pouch reaches the vulva, it becomes conoidal, and begins to protrude during pains, changing its shape according to the requirements, in much the same manner as in dilating the cervix and os; and as it protrudes more and more, the fourchette descends to within about half an inch, or less, of the anal orifice. The perineum thus, instead of being bulged and stretched to form a thin cap over the presenting part, is stretched only in the direction of its transverse diameters; the transverse muscular fibers, instead of being farther separated with each pain, as when the head is the dilator, are brought nearer together, and hence, instead of being ruptured separately and easily, can be ruptured only as a solid mass, and with proportionate difficulty.

The posterior wall of the vagina, instead of being ballooned, is but slightly stretched along its antero-posterior diameter, and hence is not liable to that attenuation or transverse rupture which makes a sort of pouch or pocket for the child's shoulder to catch in and tear through the perineum.

There are other reasons for keeping the membranes intact: malposition of the head in the pelvis can more often be determined before rupture, and be corrected later and with greater facility. The body of the child cannot easily be grasped by irregular or tonic contraction of the uterus, as it occasionally is after rupture, when the parts of the child impinge upon the interior of the uterus—a condition of affairs which, when present in only a slight form, sometimes renders impossible the rotation of the occiput forward without disastrous twisting of the child's neck. The cord is much less liable to prolapse, and, if prolapsed, can be more easily made to slip back into the uterus.

After the membranes have once protruded through the vulva, they are, of course, liable to be ruptured with any strong pain, and if they do rupture before complete dilation, there will be a liability to some contraction of the vulva before the head can descend to take the place of the membranes. In such instances there is, of course, some delay in the delivery after their rupture. After having ruptured them too soon in a number of instances, I now wait until the head threatens to come through with the membranes intact, and, in order to keep them so until then, I advise my strong patients not to bear down much, or else give a little chloroform with each pain. We can usually, by thus diminishing the pressure, delay rupture long enough for the head to be delivered with or between the succeeding pain or two. The uterus not having been long in direct contact with the fœtus, the least possible injury will occur to each; the vagina will have suffered neither from dragging down nor friction, nor will the hemorrhoidal circulation be long interfered with. But above all, the perineum will have been taken care of, and Goodell's riddle of the sphinx be solved.

Whenever the membranes rupture before their office of dilating the vagina or vulva is completed, a strong or covered rubber-bag may be introduced and then inflated with water or air, until it fills the lower unoccupied part of the vagina, so as to be made to dilate the vulva, during pains, somewhat after the manner of the membranes themselves. When a rubber-bag is not at hand, the vulva can be partly dilated by two or three fingers placed in the vagina and pressed backwards gently, but firmly, with each pain, and the capping of the head by a membranous perineum, wherein lies the chief danger to its integrity, be prevented.

3100 Forest Ave.

## Figures and Tables

**Figure f1:**
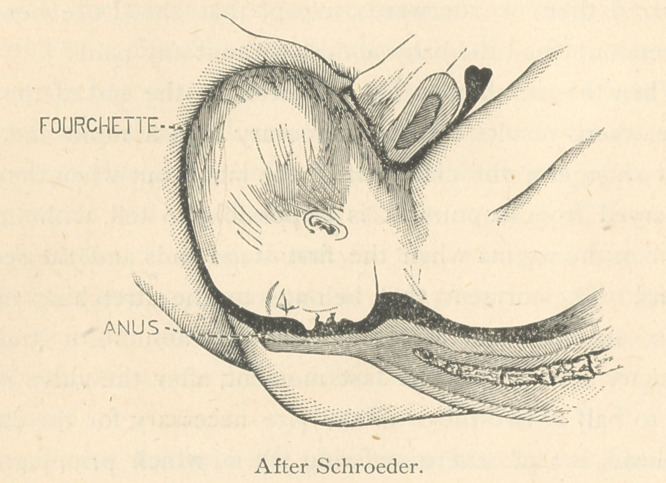


**Figure f2:**